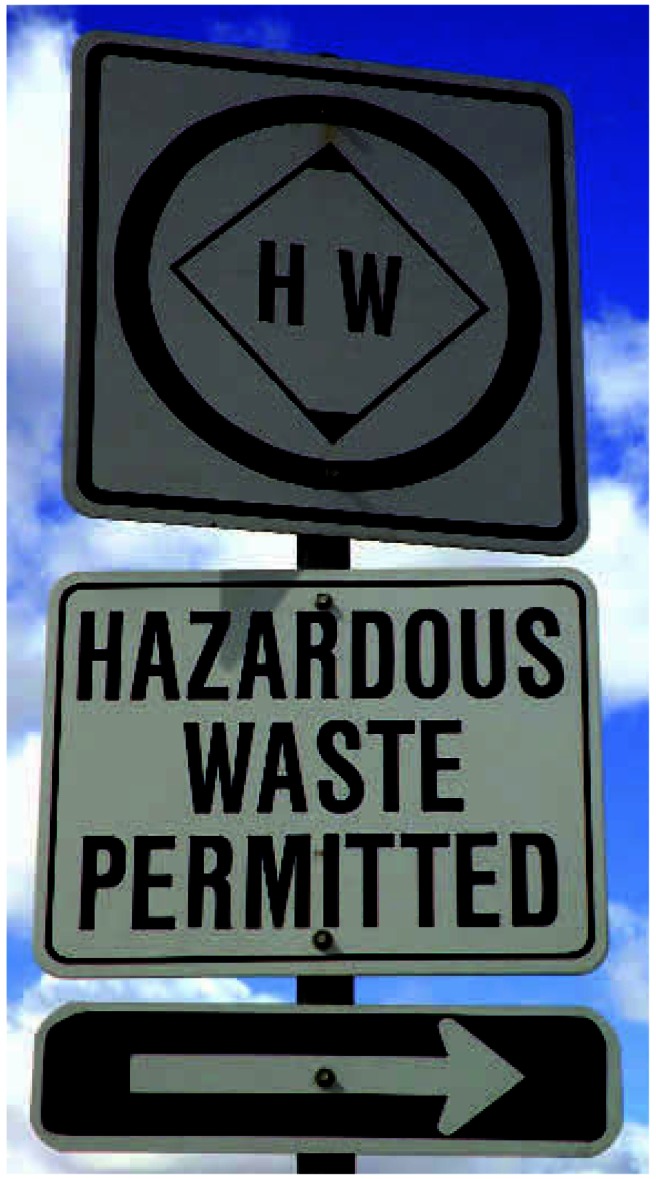# Toxic Neighbors?: Fetal Death Risk Near Hazardous Waste Sites

**DOI:** 10.1289/ehp.115-a263a

**Published:** 2007-05

**Authors:** Julia R. Barrett

The health effects associated with residential proximity to hazardous waste sites are uncertain, and findings on potential links between prenatal exposure to environmental toxicants and outcomes such as miscarriage are mixed. A recent exploratory study in Washington State finds no evidence for an overall association between hazardous waste sites and occurrence of fetal death, but pesticide-containing sites may be an exception **[*EHP* 115:776–780; Mueller et al.]**.

Using state health department records, researchers examined the occurrence of fetal death, defined as pregnancy loss after 20 weeks’ gestation, against the straight-line distance between a mother’s home and the nearest hazardous waste site. The team used ten live births for each fetal death as controls and considered several factors that could affect pregnancy outcome, such as maternal smoking, alcohol consumption, age, medical conditions, and socioeconomic status. Between 1987 and 2001, the state recorded 7,054 fetal deaths; the team located maternal residences for 5,302 cases and 61,455 controls.

Hazardous waste sites were characterized according to type of contaminant (solvents, metals, pesticides, or radioactive substances) and type of contaminated medium (air, water, or soil). Sites were also ranked as “high priority” or “low priority” depending on their potential hazard to public or environmental health.

Maternal characteristics more common among women who experienced a fetal death included being unmarried and older than 35, having less than a high school education, drinking alcohol during pregnancy, and receiving government-funded medical assistance. Mothers who experienced fetal death were also more often of nonwhite race/ethnicity and less likely to have had a previous pregnancy or birth. In general, no association was seen between hazardous waste site proximity and fetal death. However, subanalysis by contaminant type showed a small but significant increase in fetal deaths within five miles of pesticide-contaminated sites, with a slightly increased risk with each mile nearer such waste sites. Subanalysis by priority type revealed a slight but nonsignificant increase in fetal death for mothers within two miles of a high-priority site.

The authors describe several study limitations. No actual toxicant exposure measurements were available, potential occupational exposures and their duration were unknown, and fetal deaths and pertinent maternal health information may have been underreported. The findings do not negate the need for waste site remediation, however, and in light of other research showing health risks linked to prenatal pesticide exposure, the authors recommend that more attention be paid to pesticide-contaminated sites.

## Figures and Tables

**Figure:**